# Chinese Herbal Products for Non-Motor Symptoms of Parkinson’s Disease in Taiwan: A Population-Based Study

**DOI:** 10.3389/fphar.2020.615657

**Published:** 2021-01-27

**Authors:** Chien-Hung Lin, Hsienhsueh Elley Chiu, Szu-Ying Wu, Shih-Ting Tseng, Tzu-Chan Wu, Yu-Chiang Hung, Chung Y. Hsu, Hsuan-Ju Chen, Sheng-Feng Hsu, Chun-En Kuo, Wen-Long Hu

**Affiliations:** ^1^ Department of Chinese Medicine, Kaohsiung Chang Gung Memorial Hospital and Chang Gung University College of Medicine, Kaohsiung, Taiwan; ^2^ Department of Nursing, Meiho University, Pingtung, Taiwan; ^3^ Department of Sports Medicine, Kaohsiung Medical University, Kaohsiung, Taiwan; ^4^ School of Chinese Medicine for Post Baccalaureate, I-Shou University, Kaohsiung, Taiwan; ^5^ Graduate Institute of Clinical Medical Science, China Medical University, Taichung, Taiwan; ^6^ Management Office for Health Data, China Medical University Hospital, Taichung, Taiwan; ^7^ College of Medicine, China Medical University, Taichung, Taiwan; ^8^ Graduate Institute of Acupuncture Science, China Medical University, Taichung, Taiwan; ^9^ Department of Chinese Medicine, China Medical University Hospital, Taipei, Taiwan; ^10^ Kaohsiung Medical University College of Medicine, Kaohsiung, Taiwan; ^11^ Fooyin University College of Nursing, Kaohsiung, Taiwan

**Keywords:** Chaihu-Jia-Longgu-Muli-Tang, Chinese herbal products, Chinese medicine, Parkinson’s disease, *Uncaria tomentosa (Willd. ex Schult.) DC.*

## Abstract

**Objective:** Combinations of Chinese herbal products (CHPs) are widely used for Parkinson’s disease (PD) in Taiwan. Thereby, we investigated the use of CHPs in patients with PD.

**Methods:** This study was a population-based cohort study that analyzed the data of patients with PD from the National Health Insurance Research Database. A total of 9,117 patients were selected from a random sample of one million individuals included in this database. We used multiple logistic regression models to estimate the adjusted odds ratios of the demographic factors and analyzed the formula and single CHPs commonly used for PD.

**Results:** Traditional Chinese medicine users were more commonly female, younger, of white-collar status, and residents of Central Taiwan. Chaihu-Jia-Longgu-Muli-Tang was the most commonly used formula, followed by Ma-Zi-Ren-Wan and then Shao-Yao-Gan-Cao-Tang. The most commonly used single herb was *Uncaria tomentosa* (Willd. ex Schult.) DC., followed by *Gastrodia elata* Blume and then *Radix et Rhizoma Rhei* (*Rheum palmatum* L., *Rheum tanguticum* Maxim*.* ex Balf., *and Rheum officinale* Baill.). Chaihu-Jia-Longgu-Muli-Tang and *U. tomentosa* (Willd. ex Schult.) DC*.* have shown neuroprotective effects in previous studies, and they have been used for managing non-motor symptoms of PD.

**Conclusion:** Chaihu-Jia-Longgu-Muli-Tang and *U. tomentosa* (Willd. ex Schult.) DC*.* are the most commonly used CHPs for PD in Taiwan. Our results revealed the preferences in medication prescriptions for PD. Further studies are warranted to determine the effectiveness of these CHPs for ameliorating the various symptoms of PD, their adverse effects, and the mechanisms underlying their associated neuroprotective effects.

## Introduction

Parkinson’s disease (PD) results primarily from the loss of dopaminergic neurons in the substantia nigra. Treatment for PD includes drugs such as monoamine oxidase type B inhibitors, dopamine agonists, and levodopa. These agents can increase dopaminergic effects but can also result in side-effects, such as nausea, somnolence, dizziness, and headaches. More serious adverse reactions are common in the elderly, including confusion, hallucinations, delusions, agitation, psychosis, and orthostatic hypotension (Spindler and Tarsy, 2019). Levodopa is the most commonly used treatment for PD and is usually co-administered with an aromatic L-amino acid decarboxylase inhibitor to increase bioactivity, since levodopa is rapidly metabolized to dopamine by peripheral aromatic L-amino acid decarboxylase and catechol-O-methyltransferase (Rocha et al., 2017). However, it cannot penetrate the blood–brain barrier to act on the substantia nigra. Therefore, studies are currently seeking to elucidate methods to increase the levodopa levels in the brain, inhibit peripheral levodopa metabolism, and minimize the prevalence of side-effects (Ferreira et al., 2015).

Due to the limitations and side-effects of conventional therapy, patients often seek complementary and alternative medicine (CAM) (Han et al., 2017). In recent decades, the use of CAM for various diseases has increased among all adult age groups, including the elderly (Clarke et al., 2015). Previous studies have estimated the prevalence of CAM use for PD to be 25.7–76%, with survey response rates of 81–100%. Frequently utilized forms of CAM included acupuncture, massage, herbs, and vitamins/health supplements; these therapies were mainly used to improve the motor symptoms of PD (Wang et al., 2013b). Research has shown that Chinese herbal products (CHPs) have a high efficacy against PD, both in clinical trials and animal studies (Han et al., 2017). Despite the mostly positive attitude of the public toward traditional Chinese medicine (TCM), its clinical indications for specific diseases remain controversial (Hasan et al., 2010; Medagama and Bandara, 2014; Setty and Sigal, 2005). Research on the therapeutic effects of CAM may currently be restricted by small sample sizes or the lack of well-designed randomized controlled trials (Han et al., 2017).

The National Health Insurance (NHI) Research Database (NHIRD), which is widely used in Taiwan, provides a platform for understanding the utilization of CHPs by licensed TCM doctors. In this study, we analyzed the NHIRD to determine CHP utilization patterns for PD. The results may provide valuable information regarding the pattern of CHP prescription by TCM physicians, thereby improving PD treatment and forming the basis for subsequent pharmacological studies.

## Methods

### Data Source

The NHI was established on March 1, 1995, and provides medical health care to 23 million residents in Taiwan. The coverage of NHI reached 99% by the end of 2014 and therefore represents most of the medication prescription patterns in Taiwan. Each individual has a unique personal identification number in this database.

For this study, data were extracted from the Longitudinal Health Insurance Database (LHID) 2000, a subset of the NHIRD that contains all medical reimbursement claims made under the NHI program for one million enrollees from 2000 to 2011. The NHI reported that there were no significant biases in sex or age distribution in the LHID 2000 recruitment. The data used were provided by the Taiwan National Health Research Institute and authorized by the Ministry of Health and Welfare, which manages the NHI claims. These are the most recent data available. The study was approved by the Institutional Review Board of China Medical University and Hospital (CMUH104-REC2-115).

### Study Subjects

**FIGURE 1:**
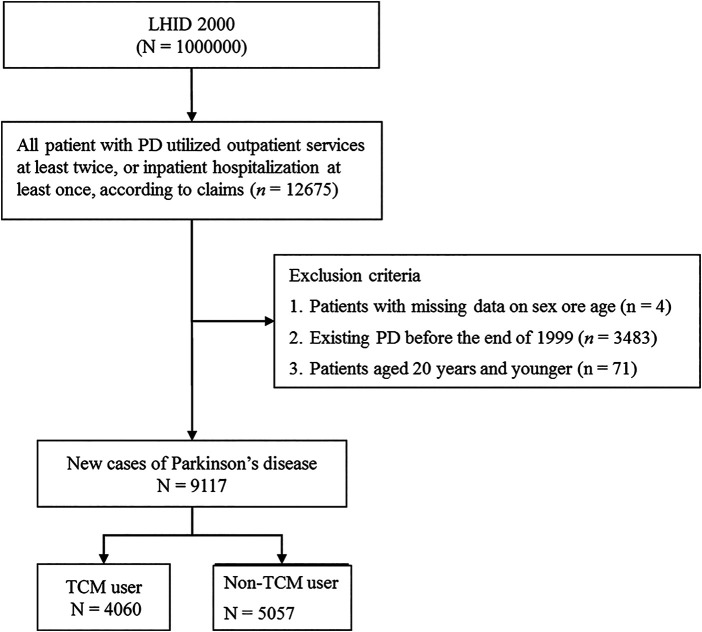
Flowchart of recruitment of subjects from LHID 2000 during 2000–2011 in Taiwan. Abbreviation: TCM, traditional Chinese medicine.

From the random sample of one million individuals enrolled in the LHID 2000, we extracted the data of patients diagnosed with PD (ICD-9-CM332, based on the International Classification of Diseases, ninth revision, clinical modification (ICD-9-CM), between 2000 and 2011. All patients with PD utilized the outpatient services at least twice or inpatient hospitalization at least once, according to the database claims (n = 12,675). We excluded patients with missing data for sex and age (n = 4), patients with existing PD diagnoses before the end of 1999 (n = 3,483), and patients aged ≤20 years (n = 71). There were 9,117 new PD cases, including TCM (n = 4,060) and non-TCM users (n = 5,057). TCM users were those who visited a TCM clinic at least once during the study period. Non-TCM users were those who had never visited a TCM clinic after the initial PD diagnosis (Figure 1).

The NHI reimburses two types of Chinese herbal remedies: Chinese single herbs and Chinese herbal formulae. Each Chinese herbal formula is a combination of two or more Chinese single herbs in strict proportions, as per the TCM literature. We analyzed the prescription patterns for PD according to these two types of herbal remedies and their usage rates.

The following sociodemographic data were extracted from the NHIRD: sex, age, occupational status, and residential area. Occupational status was divided into three categories: office workers (white collar), manual workers (blue collar), and others. The residential areas of the population were based on the district branches of the National Health Insurance Administration, consisting of Northern Taiwan, Central Taiwan, Southern Taiwan, Eastern Taiwan, and offshore islands.

### Study Analysis

TCM user and non-TCM user data (including age, sex, occupational status, and residential areas) are described as means and standard deviations. Odds ratios (ORs) and 95% confidence intervals (CIs) were used to determine which group might have a greater tendency to use TCM, based on multiple logistic regression models. We used adjusted ORs to address potential sources of bias in the use of TCM. We set statistical significance at *p* < 0.05 (two-tailed). The TCM prescription patterns for PD were analyzed according to the frequency of Chinese single herbs and Chinese herbal formulae used. All statistical analyses were run in Statistic Analysis System (SAS) software, version 9.3 (SAS Institute, Cary, NC).

## Results

### Demographic Characteristics

Among the 9,117 patients diagnosed with PD during the period 2000–2011, there were 4,060 TCM users and 5,057 non-TCM users; of the former, 434 (10.7%) used TCM, 3,578 (88.1%) used drugs for PD, and 375 (9.2%) used TCM in combination with drugs for the treatment of PD. Sex, age, occupational status, and residential areas are factors that may affect the use of TCM. Females were more likely to use TCM than males (51.1% vs. 48.9%; adjusted OR = 1.15). The mean age of TCM users (67.2 years) was lower than that of non-TCM users (72.8 years). Most of the TCM users were older than 60 years (77.1%). Younger patients were more likely to pursue TCM therapy than older patients. The adjusted ORs were 2.61 in the 20- to 29-year-old age group, 1.38 in the 30- to 39-year-old age group, 1.82 in the 40- to 49-year-old age group, and 1.63 in the 50- to 59-year-old age group. Overall, TCM users were more likely to be female, younger, of white-collar status, and living in Central Taiwan. The adjusted ORs and 95% CIs are shown in Table 1.

**TABLE 1:** Demographic characteristics and results of multiple logistic regression models showing the adjusted odds ratio and 95% confidence intervals for use of traditional Chinese medicine among patients with Parkinson’s disease during 2000–2011 in Taiwan.

Characteristics	Non-TCM user		TCM user		*p*-value	OR (95% CI)	
	N	%	N	%		Crude	Adjusted^a^
No. of cases	5,057		4,060				
Drugs for Parkinson’s disease	4,265	84.3	3,578	88.1			
TCM for Parkinson’s disease			434	10.7			
TCM + drugs for Parkinson’s disease			375	9.2			
Sex					<0.001		
Women	2,339	46.3	2076	51.1		1.22 (1.12–1.32)***	1.15 (1.06–1.26)**
Men	2,718	53.7	1984	48.9		1.00	1.00
Age at diagnosis of Parkinson’s disease, years					<0.001		
20–29	57	1.13	118	2.91		2.89 (2.10–3.98)***	2.61 (1.87–3.65)***
30–39	137	2.71	152	3.74		1.55 (1.23–1.96)***	1.38 (1.07–1.77)*
40–49	173	3.42	254	6.26		2.05 (1.68–2.50)***	1.82 (1.48–2.24)***
50–59	316	6.25	406	10.0		1.80 (1.54–2.09)***	1.63 (1.39–1.91)***
≥60	4,374	86.5	3,130	77.1		1.00	1.00
Mean (SD)	72.8	(13.1)	67.2	(14.3)	<0.001		
Occupational status					<0.001		
White collar	1,523	30.1	1,397	34.4		1.00	1.00
Blue collar	2,137	42.3	1,684	41.5		0.86 (0.78–0.95)**	0.90 (0.81–0.99)*
Others	1,397	27.6	979	24.1		0.76 (0.69–0.85)***	0.85 (0.76–0.95)**
Residential area					<0.001		
Northern Taiwan	1895	37.5	1,511	37.2		1.00	1.00
Central Taiwan	937	18.5	954	23.5		1.28 (1.14–1.43)***	1.40 (1.24–1.57)***
Southern Taiwan	1895	37.5	1,362	33.6		0.90 (0.82–0.99)*	0.93 (0.84–1.03)
Eastern Taiwan and offshore islands	330	6.53	233	5.74		0.89 (0.74–1.06)	0.94 (0.78–1.13)

TCM, traditional Chinese medicine; SD, standard deviation; OR, odds ratio; CI, confidence interval.

^a^ Model adjusted for sex, age (categorical), occupational status, and residential area.

*p < 0.05, **p < 0.01, and ***p < 0.001.

### Chinese Herbal Products for Parkinson’s Disease

An average of 5.65 CHPs were used in a single prescription for PD patients. Most prescriptions for PD involved 4 CHPs (22.4%) each, followed by prescriptions with 5 CHPs (16.2%) and prescriptions with 3 CHPs (12.1%). The most commonly used CHPs for PD by TCM doctors are shown in Table 2. Chaihu-Jia-Longgu-Muli-Tang (CJLMT, 2.7%) was the most commonly used formula, followed by Ma-Zi-Ren-Wan (MZRW, 2.12%), Shao-Yao-Gan-Cao-Tang (SYGCT, 1.58%), Tian-Wang-Bu-Xin-Dan (TWBXD, 1.57%), and Ping-Wei-San (PWS, 1.27%). The most commonly used single herbs were *Uncaria tomentosa* (Willd. ex Schult.) DC. (2.84%) followed by *Gastrodia elata* Blume (2.32%), *Radix et Rhizoma Rhei* (*Rheum palmatum* L., *Rheum tanguticum* Maxim. ex Balf., *and Rheum officinale* Baill.) (1.99%), *Salvia miltiorrhiza* Bunge (1.74%), and *Polygala tenuifolia* Willd. (1.55%) (Table 3).

**TABLE 2:** The top-10 formula Chinese herbal products (CHPs) prescribed by traditional Chinese medicine physicians for treating patients with Parkinson’s disease during 2000–2011 in Taiwan (total number of CHPs, *n* = 14,794).

Formula CHPs	Number	Frequency (%)
Chaihu-Jia-Longgu-Muli-Tang	400	2.70
Ma-Zi-Ren-Wan	313	2.12
Shao-Yao-Gan-Cao-Tang	234	1.58
Tian-Wang-Bu-Xin-Dan	232	1.57
Ping-Wei-San	188	1.27
Bu-Yang-Hwan-Wu-Tang	185	1.25
Tian-Ma-Gou-Teng-Yin	176	1.19
Xue-Fu-Zhu-Yu-Tang	148	1.00
Yi-Gan-San	138	0.93
Tiao-Wei-Cheng-Qi-Tang	136	0.92

CHPs, Chinese herbal products.

**TABLE 3:** The top-10 single Chinese herbal products (CHPs) prescribed by traditional Chinese medicine physicians for treating patients with Parkinson’s disease during 2000–2011 in Taiwan (total number of CHPs, *n* = 14,794).

Single CHPs	Number	Frequency (%)
*Uncaria tomentosa* (Willd. ex Schult.) DC.	420	2.84
*Gastrodia elata* Blume	343	2.32
*Radix et Rhizoma Rhei* (*Rheum palmatum* L., *Rheum tanguticum* Maxim. ex Balf.*, and Rheum officinale* Baill.)	295	1.99
*Salvia miltiorrhiza* Bunge	258	1.74
*Polygala tenuifolia.* Willd	229	1.55
*Acorus gramineus* Aiton	227	1.53
*Astragalus mongholicus* Bunge	161	1.09
*Rehmannia glutinosa* (Gaertn.) DC.	157	1.06
*Callerya dielsiana* (Harms ex Diels) P.K.Lôc ex Z. Wei and Pedley	142	0.96
*Magnolia officinalis* Rehder and E.H. Wilson	138	0.93

TCM, traditional Chinese medicine; CHPs, Chinese herbal products.

The top-three most commonly used formula CHPs of two combinations for PD were CJLMT plus MZRW (0.77%), TWBXD plus CJLMT (0.69%), and PWS plus Tiao-Wei-Cheng-Qi-Tang (0.62%) (Table 4). The top-three most commonly used single CHPs of two combinations were *U. tomentosa* (Willd. ex Schult.) DC. plus *G. elata* Blume (0.83%), *Acorus gramineus* Aiton plus *P. tenuifolia* Willd. (0.56%), and *Melia azedarach* L. plus *Fritillaria cirrhosa* D. Don (0.49%) (Table 5).

**TABLE 4:** The top-5 most used formula Chinese herbal products (CHPs) of two combinations for Parkinson’s disease during 2000–2011 in Taiwan (total number of CHPs, *n* = 14,794).

Two formula CHPs	Number	Frequency (%)
Chaihu-Jia-Longgu-Muli-Tang plus Ma-Zi-Ren-Wan	114	0.77
Tian-Wang-Bu-Xin-Dan plus Chaihu-Jia-Longgu-Muli-Tang	102	0.69
Ping-Wei-San plus Tiao-Wei-Cheng-Qi-Tang	92	0.62
Ping-Wei-San plus Wuo-Yao-Chun-Chi-San	68	0.46
Shao-Yao-Gan-Cao-Tang plus Gou-Teng-San	76	0.51

CHPs, Chinese herbal products.

**TABLE 5:** The top-5 most used single Chinese herbal products (CHPs) of two combinations for Parkinson’s disease in Taiwan during 2000–2011 (total number of CHPs, *n* = 14,794).

Two-combination single CHPs	Number	Frequency (%)
*Uncaria tomentosa* (Willd. ex Schult.) DC*. plus Gastrodia elata* Blume	123	0.83
*Acorus gramineus* Aiton *plus P. tenuifolia* Willd*.*	83	0.56
*Melia azedarach* L*. plus Fritillaria cirrhosa* D. Don	72	0.49
*Radix et Rhizoma Rhei* (*R. palmatum* L*., R. tanguticum* Maxim. ex Balf., and *R. officinale* Baill.) *plus Uncaria tomentosa* (Willd. ex Schult.) DC*.*	72	0.49
*Melia azedarach* L*. plus Aquilaria crassna* Pierre ex Lecomte	65	0.44

CHPs, Chinese herbal products.

## Discussion

We used the NHIRD to conduct a population-based cohort study to identify the prescription patterns of CHPs for patients with PD on a nationwide scale in Taiwan. This study reveals that CJLMT and *U. tomentosa* (Willd. ex Schult.) DC. are the most commonly used formula and single herb CHPs, respectively, for PD in Taiwan.

We further analyzed the factors that may affect TCM use by PD patients (Table 1). Females were more likely to use TCM treatment than males, consistent with the findings of Hung et al. in patients with ischemic stroke (Hung et al., 2015a). Furthermore, younger patients were more likely to use TCM than older individuals, concordant with the findings of Liao et al. in patients with chronic obstructive pulmonary disease (Liao et al., 2017). Thus, younger PD patients are more inclined to use TCM to treat their disease and maintain their daily activities, likely due to their aptitude in exploring their disease and its potential treatments. There has been no previous report regarding the relationship between TCM treatment and socioeconomic status; we found that blue-collar workers or those with other occupational statuses were less likely to seek TCM treatment than white-collar workers. Moreover, compared to the PD patients in Northern Taiwan, the PD patients in Central Taiwan tended to seek TCM treatments more often, concordant with the finding of Hung et al. that there are more TCM physicians and TCM clinics in this region than in other areas of Taiwan (Hung et al., 2015b). Therefore, people in Central Taiwan are more likely to seek combined treatment for various diseases, or to consider TCM first, than individuals residing in other areas in Taiwan.

In TCM theory, PD is characterized by tremors or muscle rigidity, which are caused by “Gan and Shan Yin deficiency,” and could result in malnourishment of the muscle, leading to “Gan Qi stagnation,” “Gan Yang excessive,” and “Endogenous Gan wind,” (Su et al., 2016). Based on the syndrome differentiation, the most common TCM syndrome patterns of PD are “Gan and Shan Yin deficiency,” “deficiency of Qi and blood,” “phlegm heat and wind stirring,” “blood stasis and wind stirring,” and “Qi stagnation and blood stasis” (Chen et al., 2017). CHPs are used depending on the syndrome differentiation. For example, Liu-Wei-Di-Huang-Wan is used for Gan and Shan Yin deficiency, and Tian-Ma-Gou-Teng-Yin is used for phlegm heat and wind stirring (Lu et al., 2016). A recent systematic review showed that several herbal extracts or their bioactive compounds had neuroprotective, neuroregenerative, and anti-oxidant properties that could reduce neuronal loss or neurodegeneration in PD models. These CHPs include *Cistanches salsa* Beck, *Scutellaria baicalensis* Georgi, *Curcuma longa* L., *Carthamus tinctorius* L., *Panax ginseng* C.A. Mey*.*, and *Silybum marianum* (L.) Gaertn*.* (da Costa et al., 2017). However, as described above, the clinical indications of CHPs for specific diseases remain controversial. In our statistical analysis, we found that the most commonly used formula CHPs and herbs are CJLMT, MZRW, SYGCT, *U. tomentosa* (Willd. ex Schult.) DC., *G. elata* Blume, and *Radix et Rhizoma Rhei* (*R. palmatum* L., *R. tanguticum* Maxim. ex Balf.*,* and *R. officinale* Baill.). The results of this study provide insights regarding personalized therapies and may form the basis for further clinical experiments and pharmacological research on the use of CHPs for the management of PD. The possible mechanisms underlying the effects of the CHPs, based on a review of the literature, are discussed below to provide insight into their pharmaceutical use.

### Commonly Used Formula Chinese Herbal Products for Parkinson’s Disease

CJLMT could harmonize Shaoyang, improve sleep quality, and alleviate depression. One of the mechanisms underlying depression includes decreased levels of monoamine neurotransmitters (norepinephrine, dopamine, and serotonin) and brain-derived neurotrophic factor in the hippocampus and prefrontal cortex. This effect can be reversed by the administration of antidepressants, such as monoamine oxidase inhibitors or selective serotonin reuptake inhibitors (Savegnago et al., 2007; Zhang et al., 2010). Saponins, which are extracted from CJLMT, were found to have antidepressant-like effects in rats; they increased the concentration of serotonin and promoted the expression of brain-derived neurotrophic factor in both the prefrontal cortex and hippocampus (Li et al., 2012). Moreover, an increase in the number of apoptotic cells in the hippocampus and cortex was found in a rat depression model, while antidepressants had an anti-apoptotic effect to protect the cells (Lucassen et al., 2004). Research has found that both saponins and antidepressants can inhibit Bax and caspase-3 (the protein associated with apoptosis) expression in the hippocampus in mice, which implies that CJLMT had an antidepressant effect via neuroprotection (Liu et al., 2010). Another rapid antidepressant is ketamine, which downregulates the N-methyl-D-aspartic acid receptor, and then upregulates the signal pathway of mammalian targets of rapamycin and α-amino-3-hydroxy-5-methyl-4-isoxazolepropionic acid receptors in the prefrontal cortex and brain-derived neurotrophic factor expression in the hippocampus. Research has shown that CJLMT can downregulate the N-methyl-D-aspartic acid receptor expression, thus resulting in a rapid antidepressant effect similar to that of ketamine (Wang et al., 2019).

The second most commonly used formula was MZRW, which is described to treat constipation in Shang Han Lun, and was effective against functional constipation in previous clinical trials (Zhong et al., 2013). Patients with PD have decreased phasic rectal contractions, weak abdominal wall contraction, paradoxical anal sphincter contraction during defecation, and defects in enteric nervous system dopaminergic neurons (Feldman et al., 2016). MZRW contains bowel-stimulating components, including aloe emodin, rhein, emodin, hesperidin, and paeoniflorin (Hu et al., 2015). Rhein and aloe emodin, extracted from *Radix et Rhizoma Rhei* (*R. palmatum* L., *R. tanguticum* Maxim. ex Balf., and *R. officinale* Baill.), were applied in a constipation rat model. The *C*_max_ and AUC of emodin in constipated rats were about ten times those of normal rats, while the *t*_1/2_ was remarkably decreased. However, a significant decrease in the AUC values for aloe emodin and rhein was observed in constipated rats compared to normal rats. Significant differences in the main pharmacokinetic parameters were found in normal and constipated rats. The study suggested that rhein and aloe emodin directly affect intestinal cell membranes, whereas emodin indirectly affects bowel movement through adjustment of the nervous system (Gong et al., 2015). Hesperidin and paeoniflorin, extracted from *Citrus aurantium* L. and *Paeonia lactiflora* Pall., respectively, have also been shown to stimulate gastrointestinal movement via the H1 histamine receptor (Fang et al., 2009).

SYGCT is typically used for Gan dysfunction, muscle cramping, spasticity, or tremors, according to Chinese medical theory. *Paeonia lactiflora* Pall. has antispastic and analgesic effects, whereas *Glycyrrhiza glabra* L. has analgesic and anti-inflammatory effects (Takao et al., 2015). SYGCT may normalize intracellular and extracellular potassium balance by inhibiting the ultra-rapid delayed rectifier potassium current and reducing potassium efflux, while the sodium-potassium pump promotes potassium influx into myofibers. Consequently, excess potassium may be reduced in the external space of myofibers. Thus, SYGCT may balance intracellular and extracellular potassium levels, and the resulting reduction in potassium levels in the external space of myofibers could alleviate muscle pain (Suganami et al., 2014). One of the components of *Glycyrrhiza glabra* L., licochalcone A, has neuroprotective as well as anti-inflammatory effects and prevents the reduction of dopaminergic neurons in PD models by inhibiting microglia-mediated neuroinflammation (Huang et al., 2017). Paeoniflorin, another bioactive component of *Paeonia lactiflora* Pall., reduces the acidosis-induced accumulation of α-synuclein and promotes autophagic degradation of α-synuclein by regulating both the expression and activity of acid-sensing ion channels, which are ligand-gated cation channels that respond to acidic stimuli (Sun et al., 2011). α-Synuclein aggregation is associated with PD pathophysiology and results in intracellular oxidative stress, inflammation, and apoptosis; thus, *Paeonia lactiflora* Pall. protects against α-synuclein cytotoxicity (Shah et al., 2014).

TWBXD is commonly used for insomnia in TCM practice. A systematic review showed the promising effect of TWBXD regarding alleviating insomnia (Yang et al., 2019). PWS is a commonly used medication for gastrointestinal disorders in TCM practice. The mechanisms of Western medicines for the treatment of functional dyspepsia include acid suppression, gastric mucosa protection, gastric motility promotion, and anti-*Helicobacter pylori* activity. Both clinical and laboratory research studies indicate that liver stagnation and spleen deficiency are the main syndromes of functional dyspepsia. Substance P is one of the most important brain–gut peptides in mammals and is composed of 11 amino acids. It is mainly distributed in the central nervous system, spinal cord dorsal root, and ENS. It promotes gastrointestinal peristalsis, protects the gastrointestinal epithelium to repair the impaired gastrointestinal mucosa, and causes gastric and intestinal mechanical hypersensitivity (Lin et al., 2012). PWS had therapeutic effects in rats with functional dyspepsia by influencing both brain–gut substance P and vasoactive intestinal peptide levels (Du et al., 2018).

### Commonly Used Single Chinese Herbal Products for Parkinson’s Disease

*U. tomentosa* (Willd. ex Schult.) DC*.* was the most commonly used single herb for PD treatment; it relieves “Gan Yang excessive” and “endogenous Gan wind” according to Chinese medical theory. *U. tomentosa* (Willd. ex Schult.) DC*.* has protective effects on dopaminergic neurons by reducing reactive oxygen species (ROS) generation; it increased glutathione levels and inhibited caspase-3 activity in a PD cell model (Shim et al., 2009).

The second most commonly used single herb was *G. elata* Blume. *G. elata* Blume decreased L-3,4-dihydroxyphenylalanine (L-DOPA)-induced dyskinesia in a PD mouse model by inhibiting phosphorylated extracellular regulated protein kinases (pERK) and FBJ murine osteosarcoma viral oncogene homolog B (FosB) expression, which are abnormally activated by long-term use of L-DOPA (Doo et al., 2014). *G. elata* Blume ameliorated the rotation behavior in PD rats and enhanced the expression of tyrosine hydroxylase positive neurons in the midbrain ventral tegmental area, displaying a neuroprotective effect on tyrosine hydroxylase positive neurons (Wang et al., 2013a). Another study in a cell model of PD revealed that a compound isolated from *G. elata* Blume had neuroprotective effects by reducing apoptosis and oxidative stress via activation of the nuclear factor erythroid 2-related factor 2 (Nrf2)/anti-oxidant response element (ARE)/heme oxygenase-1 (HO-1) signaling pathway (Huang et al., 2016).

*Radix et Rhizoma Rhei* (*R. palmatum* L., *R. tanguticum* Maxim. ex Balf*.,* and *R. officinale* Baill.), which is the third most commonly used single herb for PD, is used for constipation, jaundice, ulcers, and hemorrhages in TCM. It has been shown to exert anti-neuro-inflammatory effects by downregulating the mitogen-activated protein kinase and nuclear factor kappa B pathway, decreasing nitric oxide (NO) and ROS formation and inhibiting lipopolysaccharide-induced neuroinflammation in an animal study (Hwang et al., 2018). Rhein and aloe emodin are components with effects on bowel movements, as stated above.

*Salvia miltiorrhiza* Bunge is commonly used for dysmenorrhea, irregular menstrual cycles, ecchymosis, and insomnia in TCM practice. *S. miltiorrhiza* Bunge was found to inhibit α-synuclein aggregation both *in vitro* and in a PD model (Ji et al., 2016). Moreover, one of the effective components, tanshinone IIA, exerts neuroprotective effects by reducing the expression of lactaldehyde reductase (NADPH) oxidase and inducible nitric oxide synthase, which could further prevent nigrostriatal dopaminergic neuron loss (Ren et al., 2015).

*P. tenuifolia* Willd*.* is commonly used for insomnia and palpitation in TCM practice. *P. tenuifolia* Willd*.* exhibits anti-oxidant and anti-apoptotic activity by inhibiting ROS and NO production, which protected dopaminergic neurons in the substantia nigra and striatum against toxicity in a mouse study (Choi et al., 2011). The effects and mechanisms of the top-5 formula and single CHPs are listed in Table 6.

**TABLE 6:** Possible mechanisms underlying the effects of frequently used Chinese herbal products (CHPs) for Parkinson’s disease, ranked by prevalence of use.

Formula CHPs	Components	Mechanisms or effects
Chaihu-Jia-Longgu-Muli-Tang	*Bupleurum chinense* DC*.*, *Zingiber officinale* Roscoe, *Scutellaria baicalensis* Georgi, *Panax ginseng* C.A. Mey*.*, *Cinnamomum cassia* (L.) J.Presl, *Pinellia ternata* (Thunb.) Makino, *Radix et Rhizoma Rhei* (*R. palmatum* L*., R. tanguticum* Maxim. ex Balf*.,* and *R. officinale* Baill.), *Ziziphus jujuba* Mill*.*	CJLMT is commonly used for insomnia and depression in TCM practice.
		CJLMT reduced monoaminergic neurotransmitter system signaling and enhanced the expression of brain-derived neurotrophic factor in an animal study Li et al. (2012).
		Saponin, a component extracted from CJLMT, decreases expression of Bax and caspase-3 proteins in the hippocampus, thereby preventing neuronal apoptosis due to stress Liu et al. (2010).
		The antidepressant effect of CJLMT is probably due to reversal of the abnormal expression of N-methyl-D-aspartate and α-amino-3-hydroxy-5-methyl-4-isoxazolepropionic acid receptor in the prefrontal cortex according to a mouse study Wang et al. (2019).
Ma-Zi-Ren-Wan	*Cannabis sativa* L., *Citrus aurantium* L., *Magnolia officinalis* Rehder and E.H.Wilson, *Radix et Rhizoma Rhei* (*R. palmatum* L*., R. tanguticum* Maxim. ex Balf.*, and R. officinale* Baill.), *Prunus armeniaca* L*.*, and *Paeonia lactiflora* Pall*.*	MZRW is commonly used for constipation in TCM practice.
		Rhein, emodin, and aloe emodin affected intestinal cell membranes and bowel movement by modulating the bowel nervous system to relieve constipation in an animal study Gong et al. (2015).
		Hesperidin and paeoniflorin can stimulate gastrointestinal movement via the H1 histamine receptor Fang et al. (2009).
Shao-Yao-Gan-Cao-Tang	*Paeonia lactiflora* Pall*.* and *Glycyrrhiza glabra* L*.*	SYGCT is commonly used for pain control or rigidity in TCM practice.
		*Paeonia lactiflora* Pall. has antispastic and analgesic effects, whereas *Glycyrrhiza glabra* L*.* has analgesic and anti-inflammatory effects Takao et al. (2015). SYGCT may balance intracellular and extracellular potassium levels, resulting in reduction of potassium levels in the external space of myofibers, which can result in improvement of muscle pain Suganami et al. (2014).
		Licochalcone A has neuroprotective and anti-inflammatory effects and prevents the reduction of dopaminergic neurons in animal PD models by inhibiting microglia-mediated neuroinflammation Huang et al. (2017).
		Paeoniflorin promotes autophagic degradation of α-synuclein by regulating the expression of acid-sensing ion channels Sun et al. (2011).
Tian-Wang-Bu-Xin-Dan	*Asparagus cochinchinensis* (Lour.) Merr., *Panax ginseng* C.A. Mey*., Scrophularia ningpoensis* Hemsl., *S. miltiorrhiza* Bunge*, P. tenuifolia* Willd*., Platycodon grandiflorus* (Jacq.) A.DC., *Angelica sinensis* (Oliv.) Diels*, Schisandra chinensis* (Turcz.) Baill., *Liriope spicata* Lour., *Platycladus orientalis* (L.) Franco*,* and *Rehmannia glutinosa* (Gaertn.) DC*.*	TWBXD is commonly used for insomnia in TCM practice.
		A systematic review showed the promising effect of TWBXD in alleviating insomnia Yang et al. (2019).
Ping-Wei-San	*Magnolia officinalis* Rehder and E.H.Wilson, *Citrus aurantium* L*.*, *Atractylodes lancea* (Thunb.) DC., and *Glycyrrhiza glabra* L*.*	PWS is a common medication for gastrointestinal disorders in TCM practice.
		PWS had therapeutic effects in rats with functional dyspepsia by influencing brain–gut substance P and vasoactive intestinal peptide Du et al. (2018).
Single CHPs		
Uncaria	*Uncaria tomentosa* (Willd. ex Schult.) DC.	*Uncaria tomentosa* (Willd. ex Schult.) DC*.* is commonly used for headache, dizziness, and muscle twitching in TCM practice.
		*Uncaria tomentosa* (Willd. ex Schult.) DC. had neuroprotective effects on dopaminergic neurons by inhibiting ROS generation, increasing glutathione levels, and inhibiting caspase-3 activity in a PD cell model Shim et al. (2009).
Gastrodia	*Gastrodia elata* Blume	*Gastrodia elata* Blume is commonly used for headache, dizziness, and muscle twitching in TCM practice.
		*Gastrodia elata* Blume decreased L-DOPA-induced abnormal involuntary movement in a PD mouse model by inhibiting pERK and FosB expressions Doo et al. (2014).
		*Gastrodia elata* Blume reduced apoptosis and oxidative stress by activation of the Nrf2/ARE/HO-1 signaling pathway, exerting neuroprotective effects in a cell model of PD Huang et al. (2016).
Rhubarb	*Radix et Rhizoma Rhei* (*R. palmatum* L., *R. tanguticum* Maxim. ex Balf*.,* and *R. officinale* Baill.).	*Radix et Rhizoma Rhei* (*R. palmatum* L., *R. tanguticum* Maxim. ex Balf.*,* and *R. officinale* Baill.) is commonly used for constipation, cellulitis, and ecchymosis in TCM practice.
		*Radix et Rhizoma Rhei* (*R. palmatum* L., *Rheum tanguticum* Maxim. ex Balf., and *R. officinale* Baill.) downregulated the mitogen-activated protein kinase and nuclear factor kappa B pathway, decreases nitric oxide (NO) and reactive oxygen species formation, and inhibits lipopolysaccharide-induced neuro-inflammation in an animal study Hwang et al. (2018).
		*Radix et Rhizoma Rhei* (*R. palmatum* L., *R. tanguticum* Maxim. ex Balf., and *R. officinale Baill.)* affected intestinal cell membranes and bowel movement by modulating the bowel nervous system to relieve constipation in a rat study Gong et al. (2015).
Salvia	*S. miltiorrhiza* Bunge	*S. miltiorrhiza* Bunge is commonly used for dysmenorrhea, irregular menstrual cycle, ecchymosis, and insomnia in TCM practice.
		*S. miltiorrhiza* Bunge reduces the expression of NADPH and iNOS and prevents nigrostriatal dopaminergic neurons loss Ren et al. (2015).
		*S. miltiorrhiza* Bunge inhibited α-synuclein aggregation both *in vitro* and in a PD model Ji et al. (2016).
*P. tenuifolia*	*P. tenuifolia* Willd*.*	*P. tenuifolia* Willd*.* is commonly used for insomnia and palpitation in TCM practice.
		*P. tenuifolia* Willd*.* inhibited ROS and NO production and thereby protected dopaminergic neurons in the substantia nigra and striatum against toxicity in a mouse study Choi et al. (2011).

CHP, Chinese herbal product.

In TCM theory, “Gan Qi stagnation” is one of the TCM syndromes of PD, and it can further result in depression, anxiety, or insomnia. The frequency of use of a CHP or CHP combination represents the priority of the frequency of symptoms (Ling, 2013). CJLMT can not only relieve “Gan Qi stagnation” but also improve psychiatric symptoms and constipation (Liang and Yang, 2018). PD involves both motor and non-motor symptoms (NMSs). NMSs include mood disorders, such as apathy, anhedonia, depression, cognitive dysfunction, and hallucinosis, as well as complex behavioral disorders (Lee and Andrew, 2016; Shi et al., 2017). CJLMT is used based on the syndrome differentiation of PD, and it also relieves the NMSs of PD. Hence, it is the most commonly used CHPs formula for PD. *U. tomentosa* (Willd. ex Schult.) DC. is used for "wind stirring" which results from “Gan Qi stagnation” and “Gan and Shan Yin deficiency*.*” *U. tomentosa* (Willd. ex Schult.) DC*.* is one of the major components of Tian-Ma-Gou-Teng-Yin and is used for “Gan and Shan Yin deficiency” with “wind stirring,” which improves tremors and insomnia in PD patients (Yang et al., 2017). Another study showed that the combined use of CJLMT and Zhichan decoction (the latter contains *U. tomentosa* (Willd. ex Schult.) DC.) can relieve “Gan Qi stagnation” and “Gan and Shan Yin deficiency*.*” This formula also improved insomnia, psychiatric symptoms, and tremors in PD patients (Jiang, 2019). Current research shows that *U. tomentosa* (Willd. ex Schult.) DC*.* has neuroprotective effects on dopaminergic neurons by inhibiting ROS generation, increasing glutathione levels; inhibiting caspase-3 activity (Shim et al., 2009); increasing cell viability and mitochondrial membrane potential; decreasing lipid peroxidation, intracellular ROS, and nitric oxide; and reducing the aggregation of α-synuclein (Shi et al., 2013). Hence, it is reasonable that *U. tomentosa* (Willd. ex Schult.) DC*.* is the most commonly used single CHP for PD. Our data showed that these are common PD prescriptions in Taiwan, and they may therefore be candidates for further investigations as treatments for NMSs in patients with PD.

Current research is focused on ways to increase the level of levodopa in the brain while inhibiting peripheral levodopa metabolism in order to minimize the side-effects of the medications for PD (Setty and Sigal, 2005). A meta-analysis has shown that combining TCM with dopamine-replacement therapy is generally safe and could improve the Unified Parkinson’s Disease Rating Scale score in PD patients (Zhang et al., 2015). Based on our findings of the top-5 formula and single CHPs for PD, Table 6 shows the possible mechanisms underlying the effects of frequently used CHPs for PD, ranked by prevalence of use. These CHPs may be taken into consideration as adjunctive treatment for NMSs in patients with PD and improving the side-effects of medications for PD, according to syndrome differentiation.

A few crude medicinal herbs, over-the-counter herbal treatments, health foods containing herbs, and herbs obtained without a physician’s prescription were not included in the classification surveyed in this study. Some Chinese herbal medications can be obtained from TCM pharmacies without a prescription; therefore, the frequency of CHPs usage might have been underreported in our study. However, because the NHI system has a wide coverage of TCM prescriptions, the likelihood that patients acquired substantial quantities of herbs outside of the NHI database is very small.

The NHIRD is a large, integrative database, which includes 22.60 million people out of the 22.96 million population in Taiwan. Therefore, it contained TCM prescription patterns and clinical records that were reflective of the general Taiwanese population. However, this study had several limitations. First, the effectiveness, adverse effects, and mechanisms of these CHPs for PD were not available from this study. The NHIRD could offer prescription data; however, the chart-level records, such as laboratory data and physician notes, were not available. Second, in Taiwan, approximately 5% of TCM clinics are not contracted with the NHI program; therefore, the usage rate of TCM may have been underestimated (Chang et al., 2015). Third, patients with PD present various TCM syndromes. The NHIRD did not include data that allowed differentiation between the types of TCM syndromes in PD patients and their corresponding prescriptions. Further basic research could be conducted based on the results of this study.

## Conclusion

The most commonly used formula and single CHPs identified in this retrospective cohort study have been shown to have potential neuroprotective effects in PD by exerting anti-inflammatory, anti-oxidative, and anti-apoptotic effects. They also have effects on “Gan Qi stagnation” and “wind stirring” according to the TCM syndrome differentiation. Moreover, CJLMT has therapeutic effects on constipation, insomnia, and depression, which are common NMSs of PD. TCM adjuvant therapy may improve clinical symptoms of PD and relieve the side-effects of PD medications. Further studies are warranted to investigate the underlying mechanisms and the effectiveness of CJLMT and *U. tomentosa* (Willd. ex Schult.) DC. and to verify their use for the treatment of PD or as adjunctive medication that can be used in combination with dopaminergic agents.

## Data Availability Statement

The original contributions presented in the study are included in the article/Supplementary Material; further inquiries can be directed to the corresponding authors.

## Ethics Statement

The study was approved by the Institutional Review Board of China Medical University and Hospital (CMUH104-REC2-115).

## Author Contributions

C-EK, Y-CH, and W-LH were responsible for the study concept and design. CH and H-JC contributed to the acquisition of data. C-HL, C-EK, Y-CH, and W-LH contributed to the data analysis and interpretation of data. C-HL, HC, C-EK, and W-LH drafted the manuscript. HC, S-FH, S-YW, S-TT, and T-CW critically reviewed the manuscript. All authors approved final version for publication.

## Funding

This study was supported in part by the Taiwan Ministry of Health and Welfare Clinical Trial Center (MOHW108-TDU-B-212-133004), China Medical University Hospital, Academia Sinica Stroke Biosignature Project (BM10701010021), MOST Clinical Trial Consortium for Stroke (MOST 107-2321-B-039-04), Tseng-Lien Lin Foundation, Taichung, Taiwan, and Katsuzo and Kiyo Aoshima Memorial Funds, Japan. The funders had no role in study design, data collection and analysis, decision to publish, or preparation of the manuscript.

## Conflict of Interest

The authors declare that the research was conducted in the absence of any commercial or financial relationships that could be construed as a potential conflict of interest.

## Abbreviations

 ARE, anti-oxidant response element; CAM, complementary and alternative medicine; CHP, Chinese herbal products; CI, confidence interval; CJLMT, Chaihu-Jia-Longgu-Muli-Tang; FosB, FBJ murine osteosarcoma viral oncogene homolog B; HO-1, heme oxygenase-1; iNOS, inducible nitric oxide synthase; LHID, longitudinal health insurance database; MZRW, Ma-Zi-Ren-Wan; NHI, National Health Insurance; NHIRD, National Health Insurance Research Database; NMS, non-motor symptoms; NO, nitric oxide; Nrf2, nuclear factor erythroid 2-related factor 2; OR, odds ratio; PD, Parkinson’s disease; pERK, phosphorylated extracellular regulated protein kinases; PWS, Ping-Wei-San; ROS, reactive oxygen species; SYGCT, Shao-Yao-Gan-Cao-Tang; TCM, traditional Chinese medicine; TWBXD, Tian-Wang-Bu-Xin-Dan.
